# Advancing lithium metal batteries with *in situ* polymerized PMMA-based elastomericelectrolytes†

**DOI:** 10.1039/d4sc07685k

**Published:** 2025-03-11

**Authors:** Zhengyin Yao, Zhen Liu, Kang Xia, Haoru Xie, Shiyan Xie, Peng Zhang

**Affiliations:** a School of Materials Science and Engineering, Key Laboratory for Polymeric Composite and Functional Materials of Ministry of Education, Institute of Green Chemistry and Molecular Engineering, Sun Yat-sen University Guangzhou 510275 China zhangpeng3@mail.sysu.edu.cn; b Medical Devices Research & Testing Center, South China University of Technology Guangzhou 510006 China

## Abstract

A novel denture-inspired protocol for the preparation of poly(methyl methacrylate) (PMMA)-based solid-state elastomer electrolytes for lithium metal batteries (LMBs) has been reported in this work. The combination of succinonitrile and lithium bis(trifluoromethanesulfonyl)imide (LiTFSI) as a deep eutectic electrolyte (DEE) enables efficient dissociation of Li^+^ from TFSI^−^. Additionally, by optimizing the molar ratios of DEE and MMA to 2.16 : 1, an elastomeric electrolyte with a “polymer-in-salt” structure was developed, featuring continuous pathways for fast Li^+^ transport and high ionic conductivity (*i.e.*, 0.497 mS cm^−1^ at 30 °C). The multi-level structure of the ion transport pathways was elucidated through a combination of electron microscopy, small-angle X-ray scattering and Raman spectroscopy data. Moreover, utilizing *in situ* polymerization, robust adhesion between the electrolyte and solid electrodes was achieved, facilitating efficient Li^+^ transfer and stable solid–electrolyte interface layer formation. These electrolytes demonstrate excellent compatibility and stability with high-voltage cathodes and Li anodes, as evidenced by the superior cycling performance of LMBs. These findings provide significant insights into the design and development of new solid-state polymer electrolytes, advancing the commercial application of LMBs.

## Abstract 2

Inspired by dentures, a method for preparing PMMA-based solid elastomeric electrolytes for LMBs is proposed. This method achieves strong adhesion between the electrolyte and the solid electrode, thereby promoting efficient Li^+^ transfer.

## Graphical abstract

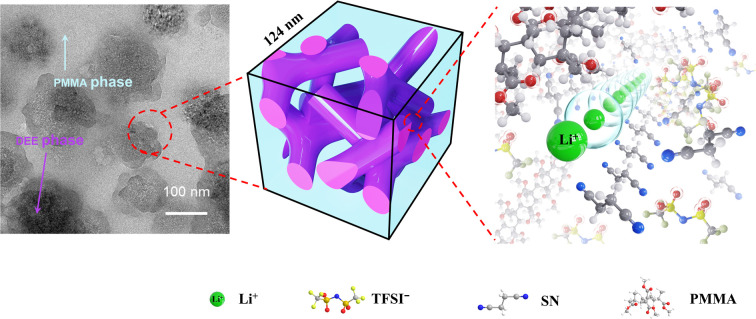

## 1 Introduction

Lithium metal batteries (LMBs) have garnered significant attention as potential next-generation energy storage devices due to their low redox potential (−3.040 V *versus* the standard hydrogen electrode) and high theoretical energy density (3860 mA h g^−1^), which surpasses that of conventional lithium-ion batteries (LIBs).^1^ LIBs, despite decades of development, are approaching their energy density and safety limits imposed by the rocking chair chemistry.^2^ Concurrently, the rapid electrification of our world drives the demand for advanced power sources to support applications in portable electronics, transportation, and grid storage. LMBs, with their ability to operate at high voltage (≥4.3 V) and provide high specific energy (more than 500 W h kg^−1^), are poised to meet these demands.^2,3^ Despite their promising characteristics, LMBs face critical challenges that hinder their practical application.^4^ Chief among these are safety concerns due to the highly reactive nature of lithium metal and performance issues such as cycling performance fading caused by lithium dendrite growth and the instability of the solid electrolyte interface (SEI).^4,5^

Addressing these challenges hinges on the choice of electrolyte materials, which play a crucial role. Solid electrolytes could offer both high energy and high-power density while establishing a more stable and safer environment for lithium metal. Among the different types of solid-state electrolytes (*i.e.*, ceramic, glass and polymer), polymer electrolytes are particularly preferred. This preference is due to the volume changes that crystalline electrodes undergo during charge/discharge cycles, making it particularly challenging to maintain stable ionic contact across interfaces with ceramic or glass electrolytes, especially at the cathode.^6^ Solid-state polymer electrolytes (SSPEs) provide numerous advantages over their ceramic and glass counterparts, including flexibility, light weight, ease of processing, and suitability for large-scale manufacturing.^7^ However, SSPEs still face critical challenges that must be addressed for large-scale commercial applications. These include low room temperature ionic conductivity, low lithium transference number, high electrolyte/electrode contact resistance due to poor wetting, insufficient electrochemical stability window, and poor stability with the Li anode.^1,8^

To address these issues, the combination of *in situ* polymerization and eutectic-based solid electrolytes to form elastomeric electrolytes has emerged as a promising approach.^9–11^ The *in situ*-formed elastomeric electrolyte ensures intimate contact between the electrolyte and the electrodes, minimizing interfacial resistance, improving mechanical stability, and facilitating fast transport of lithium ions at room temperature. Eutectic-based electrolytes can enhance ionic conductivity and stability while maintaining safety.^10^ Elastomeric electrolytes combine flexibility and resilience under mechanical stress, which can accommodate the volume changes of the electrodes during cycling, thereby maintaining contact and preventing delamination.^11^ These features are crucial for inhibiting dendrite growth and enhancing the safety of LMBs. For example, Lee *et al.* reported a class of SSPEs (SPEs) for high-energy LMBs based on an *in situ*-formed elastomer with a 3D interconnected phase of ion-conductive plastic crystals.^12^ They demonstrated that these elastomeric electrolytes successfully combined the advantages of both elastomers and plastic crystals, including high ionic conductivity, superior mechanical properties, electrochemical stability, low interfacial resistance, and a high Li^+^ transference number. Similarly, Zhang *et al.* showed that the coupling of succinonitrile (SN) and a polymer could promote the dissociation of lithium salts, resulting in excellent comprehensive electrochemical performance.^10^

Drawing inspiration from the dental industry, the *in situ* polymerization of methyl methacrylate (MMA) to create polymethyl methacrylate (PMMA)-based materials has been widely successful in the fabrication of dentures.^13,14^ Dentures require materials that exhibit excellent mechanical properties, multifunctionality, low cost and ease of processing, all of which are desirable qualities in battery electrolytes.^14^ The methodology used in dental applications, where PMMA-based materials are formed *via in situ* polymerization, provides a compelling framework for developing novel elastomeric electrolytes for LMBs. PMMA-based SSPEs have been well studied due to PMMA's excellent electrochemical and mechanical stability.^15^ For example, Martínez *et al.* reported that the PMMA matrix provided the polymer electrolyte with the highest upper limit of electrochemical stability window (5.14 V), surpassing other popular polymer materials such as polycarbonates (4.99 V), PVDF (4.94 V), PVDF-HFP (4.88 V), PAN (4.88 V), PEG (4.78 V) and PEO (4.77 V).^15^

In this work, we introduced a novel denture-inspired protocol for the preparation of PMMA-based solid-state elastomer electrolytes for LMBs. We selected a mixture of SN and lithium bis(trifluoromethanesulfonyl)imide (LiTFSI) as the deep eutectic electrolyte (DEE) due to their inherent deep eutectic hybrid characteristics. By optimizing the ratios of DEE and methyl methacrylate (MMA), we developed an elastomeric electrolyte with a “polymer-in-salt” structure. A comprehensive study was conducted to elucidate the structure–property correlations through systematic electrochemical and spectroscopic characterization studies. The coupling of PMMA and SN effectively dissociated Li^+^ from TFSI^−^, and the interconnected structure of DEE resulting from polymerization-induced phase separation facilitated efficient Li^+^ transfer at room temperature. A combination of electron microscopy, small-angle X-ray scattering, and Raman spectroscopy has been employed to characterize the Li^+^ transport pathways. Furthermore, the *in situ* polymerization process of liquid MMA ensured robust adhesion between the electrolyte and solid electrodes, leading to the formation of stable SEI layers. These layers contribute to the excellent electrochemical performance and superior cycling life of the LMBs. Our findings provide valuable insights for the design and development of new SSPEs, significantly advancing the commercial application of LMBs.

## 2 Results and discussion

**Fig. 1:**
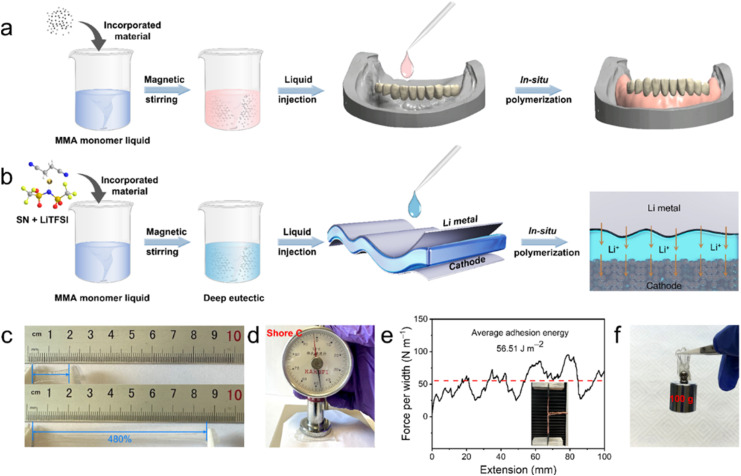
(a) Schematic illustration of the preparation process of the denture base *via in situ* polymerization of MMA. (b) Schematic illustration of the denture-base inspired preparation of *in situ* polymerized elastomeric electrolytes, consisting of PMMA, LiTFSI, SN and PEGDA. (c) Photographs of the typical elastomeric membrane (PM_2_S_3_) in the unstretched (top) and the stretched (bottom) states. (d) Photograph of the hardness test of the PM_2_S_3_ electrolyte membrane with a Shore C durometer. (e) Interfacial adhesion test of the PM_2_S_3_ membrane sandwiched between pieces of Cu foil. The red dashed line indicates the average value of force per width. (f) Photograph of the PM_2_S_3_ electrolyte membrane sticking and lifting a weight of 100 g. Note: the data in panels (c), (d) and (f) were collected in a glove box to avoid the disturbance of air and humidity.

PMMA is a synthetic polymer widely used in medical and dental applications, such as dentures and bone cement, due to its ease of processing, customizable physical and mechanical properties, cost-effectiveness, and low density.^13^ As illustrated in Fig. 1a, the liquid MMA monomer can bond well with fillers such as nanoparticles and pigments through hydrogen bonding, which is retained in the solidified PMMA *via* typical free-radical polymerization.^16^ Additionally, the introduction of a crosslinker (*e.g.*, ethylene glycol dimethacrylate, EGDMA) and an organic filler (*e.g.*, rubber) can improve the toughness and flexibility of the PMMA composites.^16^ These unique features and successful commercial applications of PMMA inspired us to design a PMMA-based SSPE (Fig. 1b) that exhibits seamless solid–solid contact, high room temperature ionic conductivity, cost-effectiveness and good stability.^17^ We employed *in situ* polymerization to achieve these objectives. The SN-LiTFSI hybrid (S) was first blended with MMA-LiTFSI (M) to form a precursor solution, followed by the precise addition of azobisisobutyronitrile (1%) and poly(ethylene glycol) diacrylate (0.5%) as the initiator and crosslinker, respectively. The homogeneous mixture was then injected into the battery assembly and polymerized at 70 °C for 12 hours, yielding PM_*x*_S_*y*_, where *x* and *y* represent the weight ratio of M to S. Adapted from dental material applications, this heat-activated free radical polymerization ensures uniform polymer formation.^18^ Notably, mixing solid LiTFSI and SN powders resulted in a liquid at room temperature (*cf.* Fig. S1 of the ESI†), due to the formation of a deep eutectic hybrid. This liquid LiTFSI-SN component endows the PMMA hybrids with an elastomeric state, as demonstrated by Lee *et al.*^12^

A similar softening strategy has been widely used in preparing solid-state elastomeric electrolytes for LMB applications.^10,12^ The improved performances of these elastomeric electrolytes, including ionic conductivity, electrochemical stability window, and interface stability, are attributed to the coupling and competition of the deep-eutectic electrolyte and polymer.^14,19^ The same principles are believed to apply to the samples shown in Fig. 1b. The elastomeric features of the as-prepared PMMA-based electrolyte can be seen in Fig. 1c and d. The electrolyte membrane sample, referred to as PM_2_S_3_ (comprising specific ratios of PMMA, LiTFSI, SN and PEGDA, detailed in the following section), was cut into a dumbbell shape and could be stretched to 480% of the original length without breaking (Fig. 1c). The elastic modulus of the electrolyte membrane was evaluated with a Shore C durometer. The Shore hardness test is a common method for qualitatively assessing and comparing the mechanical behaviour of elastomers or elastomer-like materials.^19^ The hardness value reflects the resistance of a sample to penetration by a spring-loaded needle-like indenter. Generally, the higher the hardness value, the greater the sample's resistance to indentation. Fig. 1d shows that the hardness value of the PM_2_S_3_ sample is 47C, comparable to the hardness of running shoes reported by Kleindienst *et al.* from Adidas-Salomon AG.^20^ The good anti-indentation behaviour of the PM_2_S_3_ sample was also confirmed by the nanoindentation test results (*cf.*, Fig. S2 of the ESI†). These elastomeric features indicate that the electrolyte sample should have excellent tolerance of penetration from Li dendrites. Furthermore, the soft nature of the elastomeric electrolyte may allow it to adhere well to solid electrodes, which is expected to minimize the solid–solid contact issues that are a major concern in studies of SSPEs. The adhesion behavior of the electrolyte membrane was studied using a peel test. Fig. 1e shows that the peel strength of the PM_2_S_3_ electrolyte membrane was approximately 56.51 J m^−2^, which is ten times higher than the recommended value suggested by Watson *et al.* for withstanding mechanical stress during battery fabrication and operation.^21^ This adhesion value is also higher than that of an acrylic based elastomeric electrolyte (*i.e.*, 21.5 J m^−2^) recently reported by Lee *et al.*^12^ The excellent adhesion performance of the PM_2_S_3_ electrolyte is demonstrated by its ability to lift a 100 g weight after being manually attached to the top of the weight (Fig. 1f).

**Fig. 2:**
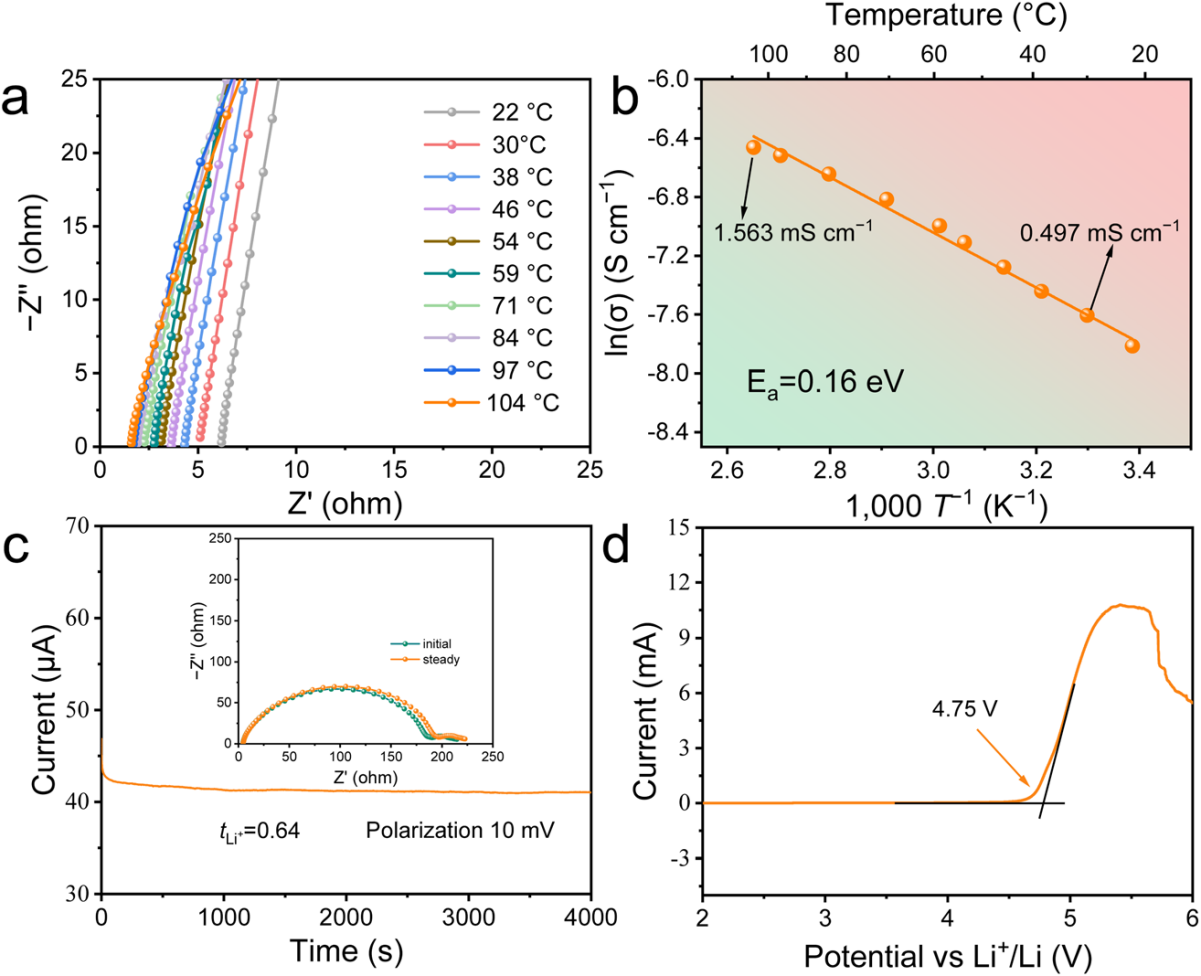
(a) The temperature dependence of electrochemical impedance spectroscopy (EIS) Nyquist plots of the PM_2_S_3_ sample measured in a symmetric SS‖SS blocked battery. (b) Arrhenius plots of the PM_2_S_3_ sample derived from the temperature-dependent ionic conductivity results in panel (a). (c) Current variation with time during polarization of a Li|PM_2_S_3_|Li battery at 30 °C with an applied DC voltage of 10 mV. (d) Linear sweep voltammetry profile of the PM_2_S_3_ sample in an SS|PM_2_S_3_|Li asymmetric battery at 30 °C.

A systematic study was undertaken to manipulate the relative contents of MMA, LiTFSI and SN to achieve optimal performance, considering room temperature ionic conductivity (*σ*_i_) and high-voltage stability. These factors were chosen based on two main considerations. First, there are well-established, clear and robust test methods for these metrics. Second, they are generally accepted as key factors for the development of SSPEs. The MMA and SN were mixed independently with LiTFSI at different molar ratios. It was determined that the optimal molar ratios for MMA-LiTFSI (denoted as M) and SN-LiTFSI (denoted as S) were 10 : 1 and 10 : 1 (*cf.*, Fig. S3 of the ESI†). The optimized samples were then mixed at different weight ratios, given that both were in a liquid state. As the SN-LiTFSI concentration increased, the *σ*_i_ of polymerized samples (*i.e.*, PM_*x*_S_*y*_, *cf.*, Fig. S4a of the ESI†) improved. With a mass ratio of MMA-LiTFSI : SN-LiTFSI = 2 : 3 (PM_2_S_3_), PM_2_S_3_ shows an impressive room temperature *σ*_i_ of 0.497 mS cm^−1^ at 30 °C. However, further increasing the SN-LiTFSI concentration in PM_*x*_S_*y*_ does not significantly improve ionic conductivity but instead decreases mechanical properties and increases flowability (*cf.*, Fig. S5 of the ESI†). The polymerized samples were characterized to determine the optimal composition of PM_2_S_3_, with *σ*_i_ = 0.497 mS cm^−1^ at 30 °C (Fig. 2a). Note: PM_1_S_4_ was not considered due to its liquid-like characteristics (Fig. S4 in the ESI†), despite exhibiting a higher *σ*_i_ value than the PM_2_S_3_ sample (Fig. S5 in the ESI†).

An in-depth study was conducted to understand the superior room temperature ionic conductivity of PM_2_S_3_ and its potential application in high-voltage LMBs. The relative molar ratio of MMA : SN : LiTFSI in PM_2_S_3_ was calculated to be 3.51 : 6.58 : 1, indicating that the amount of DEE was 2.16 times that of MMA. According to the previous studies, the high proportion of DEE endowed PM_2_S_3_ with a “polymer-in-salt” structure, which is known to enhance *σ*_i_ by forming an ionic conductive network independent of the polymer chain flexibility. The temperature-dependent ionic conductivity behaviour was examined to explore the ion transport behaviour of the PM_2_S_3_ sample. Fig. 2a shows that the internal resistance of the SS|PM_2_S_3_|SS blocked battery lies in the range of 1–6 Ω. This low internal resistance suggests that PM_2_S_3_ allows efficient ion transport due to its low bulk resistance and seamless electrolyte–electrode contact. Compared to the EIS Nyquist data of liquid-state M and S samples (∼5 Ω in Fig. S3 of the ESI†), the low internal resistance of PM_2_S_3_ could be attributed to its liquid-state M_2_S_3_ origins. On the one hand, it is well known that the DEE consisting of LiTFSI and SN shows good room-temperature ionic conductivity. On the other hand, liquid MMA can wet well with solid objects such as fillers or pigments, maintaining this characteristic post-polymerization, as demonstrated in denture applications. To confirm our hypothesis, the change in *σ*_i_ of M_2_S_3_ at 70 °C as a function of polymerization time was investigated. It was found out that M_2_S_3_ polymerization did not increase the energy barrier for Li^+^ transportation in the polymer matrix (*cf.*, Fig. S4b of the ESI†).

Fig. 2a shows that the resistance value of PM_2_S_3_ decreases monotonically with increasing temperature. To understand this phenomenon, the temperature-dependent *σ*_i_ of PM_2_S_3_ was analysed using the Arrhenius equation (Fig. 2b). The activation energy (*E*_a_) of PM_2_S_3_ was estimated to be 0.16 eV, similar to the 0.13 eV reported for *in situ* polymerized elastomeric electrolytes by Kim *et al.*^12^ They attributed the low activation energy to the well-established conductive pathways of DEE domains. This principle has also been reported by Zhou *et al.* for solid-state elastomeric electrolytes with typical “polymer-in-salt” structures.^22^ Thus, it is inferred that the “polymer-in-salt” structure helps PM_2_S_3_ establish well-connected ion-conductive pathways.

The lithium transference number (*t*_Li^+^_) of PM_2_S_3_, important for high power densities and fast charging of batteries, was then studied (Fig. 2c) using the typical Bruce–Vincent method.^23^ Based on the data in Fig. 2c, the *t*_Li^+^_ of PM_2_S_3_ was calculated to be 0.64, indicating that Li^+^ motion accounts for the majority of the total ionic conductivity. This value is higher than that of most traditional organic electrolytes (<0.5) and many SSPEs (*e.g.*, <0.3 for PEO and 0.46 for PMMA).^22,24^ The well-connected ionic conductive pathways facilitate ion transport, and SN exhibits higher complexation stability with Li^+^ compared to the ester groups in PMMA or the TFSI^−^ ions.^25^ This complexation stability is indicated by the complex formation constant (*K*_complex_) with Li^+^, representing the polymer chain's affinity for cations.^26^ Dimov *et al.* demonstrated that the *K*_complex_ of related monomers follows the order: nitrile > ketones > ethers > esters.^27^ Thus, SN partially screens the interactions between Li^+^ and ester groups in PMMA/TFSI^−^. This hypothesis aligns with the chemical composition of the electrolyte, where the molar ratio of MMA : SN : LiTFSI in PM_2_S_3_ is 3.51 : 6.58 : 1, with SN being the majority component.

Next, the high-voltage stability of PM_2_S_3_ was studied using linear sweep voltammetry and electrochemical floating methods in SS‖Li and NCM811‖Li asymmetric batteries, respectively. Fig. 2d shows that the elastomeric electrolyte is electrochemically stable up to 4.75 V. This high-voltage stability is slightly higher than the literature values for S electrolytes (4–4.5 V), indicating improved compatibility with high-voltage cathodes. The good electrochemical stability and compatibility with high-voltage cathodes were confirmed by the electrochemical floating results of NCM811‖Li (*cf.*, Fig. S7 of the ESI†), which showed a low leakage current (similar to that reported by Zhang *et al.* for the DEE hybrid) up to 4.5 V.^28^ The leakage current provides a straightforward means of assessing the oxidative stability of the elastomeric electrolyte. The slightly lower value compared to that determined by the LSV method may be due to the side reaction between PM_2_S_3_ and Li at high voltage and prolonged time. It is well known that SN has poor reduction stability against the Li anode.^10^ A similar phenomenon was reported by Lee *et al.* for SN/LiTFSI-based elastomeric electrolytes in LMBs.^12^ These findings indicate the good suitability of PM_2_S_3_ for LMBs with high-voltage cathodes.

**Fig. 3:**
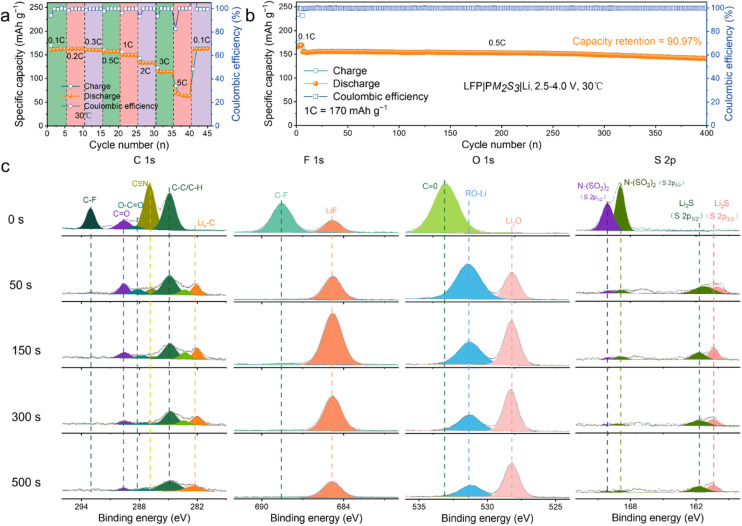
Illustration of the performance metrics of the LFP|PM_2_S_3_|Li batteries: (a) C-rate performance, (b) cycling life and (c) XPS spectra for C 1s, F 1s, O 1s and S 2p core levels of the Li anode after various Ar^+^ sputter-cleaning times. The Li anode was cycled in a Li|PM_2_S_3_|Li symmetric battery for 2000 hours.

To verify our hypothesis, PM_2_S_3_ was assembled into full LMBs with different high-voltage cathodes, *i.e.*, LiFePO_4_ (LFP), LiNi_0.8_Co_0.1_Mn_0.1_O_2_ (NCM811) and LiCoO_2_ (LCO), and their performance was evaluated in terms of capacity, rate performance and cycling life. A small amount (5 wt% of SN/MMA) of fluorinated ethylene carbonate (FEC) was added to the electrolyte to mitigate side reactions between the electrolyte and Li, as referenced in the work of Effat *et al.*^29^Fig. 3a shows the excellent rate performance of the LFP|PM_2_S_3_|Li battery. The battery delivered capacities of 163.02, 162.69, 160.14, 156.68, 149.88, 133.10, 114.92, and 69.78 mA h g^−1^ at 0.1, 0.2, 0.3, 0.5, 1, 2, 3, and 5C, respectively. Notably, the battery restored a capacity of 162.62 mA h g^−1^ when the rate was reverted to 0.1C after the rate performance evaluation, indicating good cycling stability. To further evaluate the cycling stability of the battery, it was tested at 0.5C (170 mA h g^−1^) and 30 °C. After being activated at 0.1C for 5 cycles, the battery was cycled at 0.5C within a voltage range of 2.5–4.0 V. As shown in Fig. 3b, the battery retained a capacity of 141.00 mA h g^−1^ (90.97% capacity retention) after 400 cycles, with an average coulombic efficiency (CE) of 99.85%. The good cycling performance of the battery suggests that the elastomeric electrolyte retains good contact and electrochemical stability with the electrodes. This inference is supported by the good electrochemical stability of the batteries throughout cycling, as indicated by both galvanostatic charge/discharge (GCD) and cyclic voltammetry (CV) profiles (*cf.*, Fig. S8 of the ESI†).

To further validate our hypothesis, a lithium plating/stripping experiment was conducted with a Li|PM_2_S_3_|Li symmetric battery. The battery cycled stably for more than 2000 hours with a current density and areal capacity of 0.1 mA cm^−2^ and 0.1 mA h cm^−2^ (*cf.*, Fig. S9 of the ESI†). The voltage curves showed minimal polarization over 2000 hours of cycling. Additionally, the characteristic ‘arch’ shape at the edge of the voltage profile, typically attributed to dendritic and dead Li accumulation, was absent even after repeated plating and stripping of Li metal for 2000 hours. These features indicate that PM_2_S_3_ has good compatibility with the Li anode, enabling stable Li plating and stripping cycles even at higher current densities, as evidenced in Fig. S10 of the ESI.† A similar phenomenon has been reported by Lee *et al.* in their work on DEE-based elastomeric electrolytes.^12^ According to the literature, the formation of a protective SEI layer plays a crucial role in the batteries' high cycling stability.^30^ To explore the chemical composition of the SEI layer, the cycled Li anode was analysed with X-ray photoelectron spectroscopy (XPS), and the depth profile was collected with the assistance of Ar^+^ sputter-cleaning (Fig. 3c). As indicated by the 0-minute of Ar^+^ sputter-cleaning data, the surface of the SEI was composed of an organo–mineral complex, such as the F-rich, O-rich, and S-rich compositions: C–F (688.6 eV, F 1s), Li–F (685 eV, F 1s), C=O (533.2 eV, O 1s), ROLi (531.5 eV, O 1s), Li_2_O (528.2 eV, O 1s), N–(SO_2_)_2_ (168.9 and 170.2 eV, S 2p) and Li_2_S (160.4 and 161.8 eV, S 2p).

Additionally, the C species, consisting of C–F (293.0 eV, C 1s), C=O (289.7 eV, C 1s), O–C=O (288.5 eV, C 1s), C≡N (286.7 eV, C 1s), C–C/C–H (284.8 eV, C 1s) and C–Li (282.1 eV, C 1s), demonstrated that the organic-rich content was mainly distributed at the surface of the SEI. With the increase in sputter-cleaning time from 50 to 500 s, the signal of organic components such as C≡N, C=O, C–O and C–C/C–H diminished; in contrast, the inorganic components, including Li_2_S, Li_2_O and LiF, were predominantly formed, as indicated by the increase in their XPS signal. Thus, an organic-rich outer layer and an inorganic inner layer formed in the SEI of the cycled Li anode. This SEI layer is believed to facilitate the Li^+^ transport during the stripping/plating process, inhibiting the rapid growth of Li dendrites.^31,32^ This inference was confirmed using SEM data (*cf.* Fig. S11a of the ESI†), which showed a smooth and robust surface in the SEI layer of the cycled Li anode. Additionally, the surface and cross-sectional morphology of LFP in the LFP|PM_2_S_3_|Li battery after 500 cycles at 1C and 30 °C were characterized with SEM (*cf.* Fig. S11b and S11c of the ESI†). Generally, the surface of the LFP cathode remained flat, with no noticeable cracks or delamination from the aluminium foil. This observation supports the good cycling life of the LFP|PM_2_S_3_|Li batteries.

**Fig. 4:**
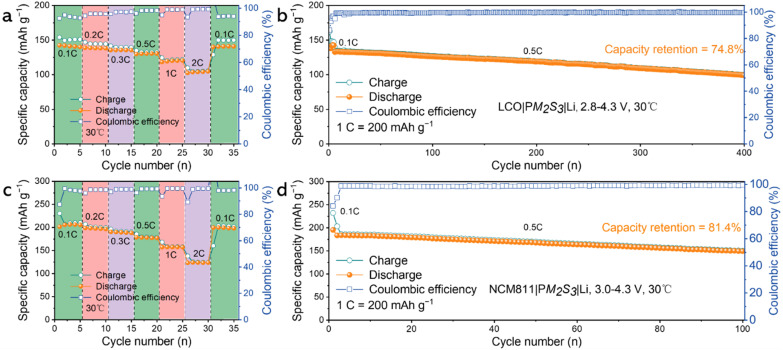
Illustration of the performance metrics of the LCO|PM_2_S_3_|Li batteries: (a) C-rate performance and (b) cycling life; and the NCM811|PM_2_S_3_|Li batteries: (c) C-rate performance and (d) cycling life.

**Fig. 5:**
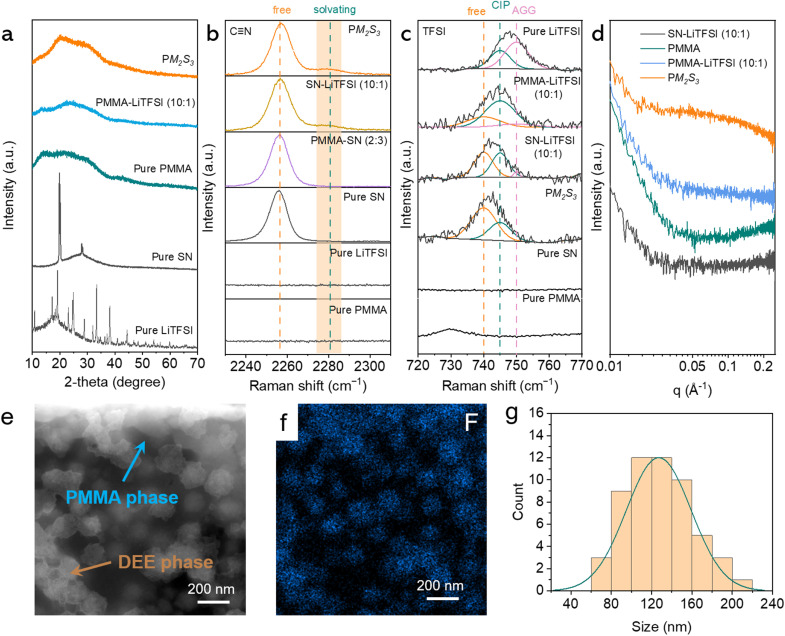
(a) X-ray diffraction data of the PM_2_S_3_ and reference samples. Raman spectra of PM_2_S_3_ and reference samples in (b) 2230–2310 cm^−1^ and (c) 720–780 cm^−1^ regions. Points and solid lines correspond to experimental data and fitting curves, respectively. (d) SAXS data of PM_2_S_3_ and reference samples. (e) HAADF-STEM and (f) EDS mapping images of PM_2_S_3_. In panel (f), the blue area indicates the F-rich region. (g) The statistical size results and their Gaussian fitting of the DEE regions, based on the data in panel (e).

To demonstrate the versatility of the elastomeric electrolytes for high-performance LMBs, we assembled and tested both LCO|PM_2_S_3_|Li and NCM811|PM_2_S_3_|Li batteries. The cut off voltage was set at 4.3 V, a generally accepted threshold value to avoid irreversible phase changes. Fig. 4a shows that the LCO|PM_2_S_3_|Li battery exhibited specific capacities of 142.4, 138.9, 135.7, 130.6, 120.6, and 104.9 mA h g^−1^ at 0.1, 0.2, 0.3, 0.5, 1, and 2C, respectively, demonstrating robust rate capacity across various current densities. When the current density was reverted to 0.1C, the specific capacity returned to its original value, indicating excellent reversibility of the LCO|PM_2_S_3_|Li battery. This was further corroborated by the cycling life measurements, as shown in Fig. 4b. The LCO|PM_2_S_3_|Li battery had an initial capacity of 132.7 mA h g^−1^, which decreased to 99.3 mA h g^−1^ with a coulombic efficiency (CE) of 99.54% at 0.5C after 400 cycles at 30 °C, resulting in a capacity retention of 74.8%. Fig. 4c shows that the NCM811|PM_2_S_3_|Li battery had specific capacities of 206.2, 198.8, 190.4, 178.6, 157.6, and 124.1 mA h g^−1^ at 0.1, 0.2, 0.3, 0.5, 1, and 2C, respectively. These values surpass those of the LCO|PM_2_S_3_|Li battery (Fig. 4a). Similar to the LCO|PM_2_S_3_|Li battery, the NCM811|PM_2_S_3_|Li battery also exhibited good cycling performance. As shown in Fig. 4d, the NCM811|PM_2_S_3_|Li battery had an initial capacity of 183.5 mA h g^−1^, which decreased to 149.4 mA h g^−1^ with a CE of 99.66% at 0.5C after 100 cycles at 30 °C, resulting in a capacity retention of 81.4%. These performances are comparable to those recently reported by Peng *et al.* based on fluorine-containing polymer frameworks.^11^ Moreover, a NCM811|PM_2_S_3_|Li pouch battery was assembled to evaluate the dimensional stability and safety of the battery. The pouch cell operated well even after being cut, folded and punctured (*cf.* Fig. S12 of the ESI†), demonstrating its good stability and excellent safety. The remarkable performances of both coin and pouch cells reveal the good electrochemical and mechanical properties of the PM_2_S_3_ electrolyte, inspiring us to explore the structure–property correlations using complementary structure characterization methods. The crystalline behaviour of the elastomer electrolyte was characterized using X-ray diffraction, a common method to analyse the structure and coordination status of lithium salt. Fig. 5a shows that PM_2_S_3_, PMMA-LiTFSI and PMMA do not exhibit crystalline signals, indicating their amorphous nature. A broad peak centered around 2*θ* = 20° was observed, attributed to the adjacent distances of the molecular segments.^33^ In contrast, crystalline peaks were found in SN and LiTFSI, consistent with their crystalline nature. It is inferred that the absence of crystalline signals for SN and LiTFSI in the elastomeric electrolyte is due to the strong molecular interactions among the components, inhibiting crystallization.

To study the molecular interactions in the PM_2_S_3_ electrolyte, Raman spectroscopy data were collected. Fig. 5b shows that neat SN has a Raman band centered at 2258 cm^−1^, attributed to the stretching band (v_2_ mode) of C≡N in free SN molecules.^34^ When SN is mixed with LiTFSI, a new band at 2282 cm^−1^ appears, indicating strong coordination among Li^+^ and SN molecules. This suggests that SN might replace TFSI^−^ to couple with Li^+^, which can be inferred from the Raman signal of the TFSI^−^ ions. Fig. 5c shows the Raman band of the TFSI^−^ ions, attributed to the vibration of TFSI^−^ ions, including S–N stretching, C–S stretching, and CF_3_ bending. According to Seo *et al.*, these bands can be divided into three peaks at 740, 745, and 750 cm^−1^, corresponding to free anions, contact ion pairs (CIPs, TFSI^−^ coordinating to a single Li^+^ cation), and aggregates (AGGs, TFSI^−^ coordinating to two or more Li^+^ cations), respectively.^35^ Based on the deconvolution analysis results, the changes in the TFSI^−^ structure as a function of mixing components were qualitatively studied by examining the relative areal ratio of these three peaks. Fig. S14 of the ESI† shows that TFSI^−^ exists only in CIP and AGG states in the LiTFSI sample. Mixing LiTFSI with PMMA or SN results in the presence of free anions, while free anions and CIPs dominate in the PM_2_S_3_ sample. This can be explained by the fact that both PMMA and SN can dissociate TFSI^−^ and Li^+^ and couple with each other to enhance the dissociation effect.

The multi-level Li^+^ transport pathways were further investigated using small-angle X-ray scattering (SAXS), high-angle annular dark-field scanning transmission electron microscopy (HAADF-STEM), and energy-dispersive X-ray spectroscopy (EDS). SAXS is a well-established technique for characterizing the microstructure in polymer hybrids.^36–38^ Notably, due to the high penetration depth of X-rays, SAXS provides statistical structural information averaged over the illuminated sample volume (approximately 1.5 mm along the beam direction in this work).^39,40^ As shown in Fig. 5d, the SAXS profile of PM_2_S_3_ exhibits a weak and bumpy peak centered around *q* = 0.05 Å^−1^. Similar scattering features have been observed in our previous studies on PMMA-g-polyisoprene/LiTFSI systems and were attributed to LiTFSI clustering.^40^ To verify this assignment, SAXS measurements were performed under identical conditions for the individual components of PM_2_S_3_, namely PMMA, PMMA/LiTFSI, and SN/LiTFSI (Fig. 5d). By comparison, the distinctive bumpy peak appears only in PM_2_S_3_, indicating a characteristic microstructure with a periodicity of 12.6 nm, calculated using 2π/*q*.

To gain further insight into the microstructure, HAADF-STEM and EDS mapping were employed. As shown in Fig. 5e, PM_2_S_3_ exhibits microphase separation, characterized by regions of varying contrast. The EDS mapping data (Fig. 5f) further confirm that the blue-colored F-rich domains correspond to TFSI^−^ from the DEE component, indicating its phase separation from PMMA. Statistical analysis based on a Gaussian fitting model estimates the average size of DEE microdomains to be 124 nm (Fig. 5g), assuming a spherical morphology. Similar phase separation behavior was recently reported by Kim *et al.*, further supporting this observation.^41,42^ By integrating SAXS and TEM findings, we attribute the 12.6 nm feature to the diameter of the interconnected DEE transport channels.

**Fig. 6:**
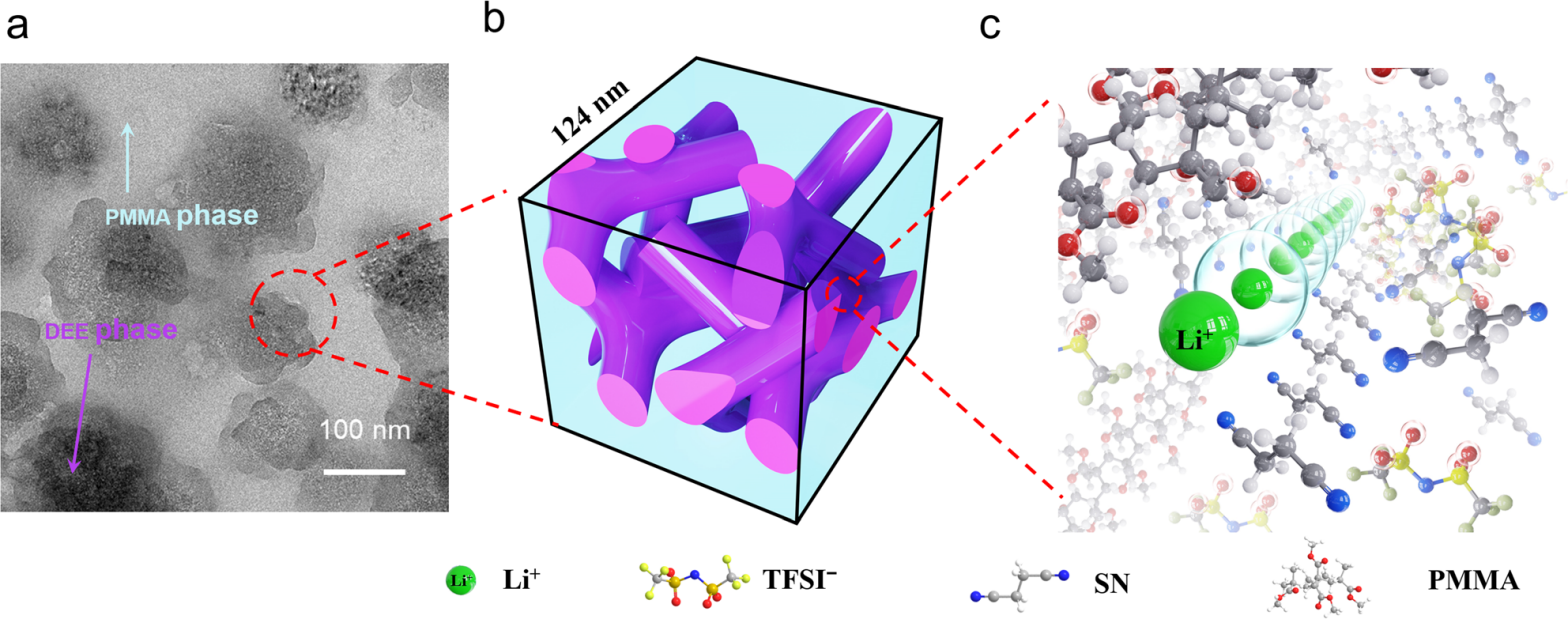
Schematically illustrating the interconnected structure of elastomeric electrolytes prepared by denture-inspired *in situ* polymerization of MMA, with pipelines consisting of SN and LiTFSI that provide fast pathways for Li^+^ transfer. (a) Transmission electron microscopy images of PM_2_S_3_. (b) Interconnected pipeline structure of PM_2_S_3_. (c) Fast pathways for Li^+^ transfer.

On the one hand, the denture-inspired *in situ* polymerization of MMA not only facilitates close and robust adhesion of the polymer electrolyte to the solid electrodes but also maintains the integrity of the DEE microdomains (size ∼124 nm, Fig. 6a and b). On the other hand, the amount of DEE is 2.16 times that of MMA, endowing PM_2_S_3_ with a “polymer-in-salt” structure. As evidenced by spectroscopy data, PMMA couples with SN to dissociate LiTFSI (Fig. 6c), forming pipelines or interconnected microdomains.^25,43^ In these pipelines (diameter ∼12.6 nm, Fig. 6b), Li^+^ ions are coupled with mobile SN molecules, supporting high room temperature ionic conductivity and a high Li^+^ transfer number.

## 3 Conclusions

In this study, we successfully demonstrate a denture-inspired protocol for the preparation of SSPEs for LMBs. The *in situ* polymerization process facilitates the robust adhesion between the electrolyte and solid electrodes that enables the Li^+^ transfer and formation of stable SEI layers. In addition, the mixture of SN and LiTFSI inherits deep eutectic hybrid features that provide fast transport of ions at room temperature. In addition, the coupling of PMMA and SN dissociate Li^+^ from TFSI^−^, which rationalizes the high Li^+^ transfer number. Moreover, by manipulating the ratios of DEE and MMA, a “polymer-salt-structure” was achieved in the PM_2_S_3_ sample. These DEEs form continuous pipelines that allow fast transport of dissociated Li^+^ and, thus, high room temperature ionic conductivity was achieved. The multi-level structure of these pipelines was revealed through a combination of electron microscopy, small-angle X-ray scattering, and Raman spectroscopy. Moreover, the elastomeric electrolytes showed good compatibility and stability with high-voltage cathodes and Li anodes, as evidenced by the good cycling lives of LFP|PM_2_S_3_|Li, LCO|PM_2_S_3_|Li and NCM811|PM_2_S_3_|Li batteries. The success of these LMBs was attributed to both the robust nature of the elastomeric electrolyte and the formation of a stable SEI layer on the surface of the Li anode. The present findings are expected to help in the design and preparation of new SSPEs towards the commercial applications of LMBs. Future work will focus on further optimizing the electrolyte composition and exploring additional performance enhancements to address remaining challenges in LMB technology.

## Data availability

All the data supporting the findings of this study are available within the article and its ESI.† Additional data related to this article can be obtained from the corresponding author upon reasonable request.

## Author contributions

P. Z. conceived the idea and directed the project. Z. Y. and K. X. proposed and designed the experiments. Z. Y. prepared the polymer electrolytes and electrodes and performed electrochemical measurements and battery tests. Z. L. collected SEM, HAADF-STEM, EDS and load-displacement data. S. X. performed Raman measurements. Z. Y. wrote the manuscript, and P. Z., Z. L. and H. X. edited the manuscript. All authors contributed to the discussion.

## Conflicts of interest

There are no conflicts to declare.

## Supplementary Material

SC-016-D4SC07685K-s001
