# Supplementary material for: Advancing lithium metal batteries with in situ polymerized PMMA-based elastomericelectrolytes
Source: Chem Sci. 2025 Mar 11;16(16):6812–21. doi: 10.1039/d4sc07685k (PMC11915133; doi:10.1039/d4sc07685k)
Supplement: SC-016-D4SC07685K-s001 [file SC-016-D4SC07685K-s001.pdf]

Supplementary Information (SI) for Chemical Science.
This journal is © The Royal Society of Chemistry 2025

Advancing Lithium Metal Batteries with In Situ Polymerized PMMA-Based Elastomeric Electrolytes

Zhengyin Yao[a], Zhen Liu[b], Kang Xia[a], Haoru Xie[a], Shiyan Xie[a], and Peng Zhang[a,*]

a. School of Materials Science and Engineering, Key Laboratory for Polymeric Composite and Functional Materials of Ministry of Education, Institute of Green Chemistry and Molecular Engineering, Sun Yat-sen University, Guangzhou 510275, China;

b. Medical Devices Research & Testing Center, South China University of Technology, Guangzhou 510006, China

*Corresponding author:

Peng Zhang, Email: zhangpeng3@mail.sysu.edu.cn

1. Experimental section

1.1 Materials

The following chemicals were used as received: Methyl methacrylate (MMA, purity 99.0%, Shanghai Macklin Biochemical Technology Co., Ltd., China), N-Methyl-2-pyrrolidone (NMP, electronic grade, purity 99.9%, Shanghai Aladdin Biochemical Technology Co., Ltd., China), Succinonitrile (SN, purity 99.0%, Shanghai Aladdin Biochemical Technology Co., Ltd., China), Lithium bis(trifluoromethanesulfonyl)imide (LiTFSI, purity 99.0%, Shanghai Aladdin Biochemical Technology Co., Ltd., China), Poly(ethylene glycol) diacrylate (PEGDA, purity 99.0%, Shanghai Macklin Biochemical Technology Co., Ltd., China), 2,2'-Azobis(2-methylpropionitrile) (AIBN, purity 99.0%, Shanghai Aladdin Biochemical Technology Co., Ltd., China), and Fluoroethylene carbonate (FEC, purity 99.0%, Shanghai Aladdin Biochemical Technology Co., Ltd., China). Additional materials, including $LiFePO_4$ (LFP, electronic grade), $LiCoO_2$ (LCO, electronic grade), $LiNi_{0.8}Co_{0.1}Mn_{0.1}O_2$ (NCM811, electronic grade), poly(vinylidene fluoride) (PVDF, Mw = 455,000 Da), Super P carbon black (electronic grade), polypropylene diaphragms (electronic grade), lithium metal foil, stainless steel (SS) plate, aluminum (Al) foil, copper (Cu) foil, nickel (Ni) foil, 2025-type coin battery components (case, gasket, spacer, wave spring, and cap), and plastic-coated aluminum soft pack (70×70 mm^2) were purchased from Guangdong Canrd New Energy Technology Co., Ltd., China.

1.2 Electrolyte preparation

The elastomeric electrolytes were prepared in an Ar-filled glove box using in situ free

radical polymerization, a common method for PMMA-based dentures. Initially, the mixtures of MMA-LiTFSI and SN-LiTFSI were studied separately to optimize their compositions for both room temperature ionic conductivity and high-voltage stability. The molar ratios for MMA-LiTFSI (10:0.5 to 10:10) and SN-LiTFSI (10:0.5 to 10:3) mixtures were optimized to 10:1 due to solubility limitations. Subsequently, the MMA-LiTFSI and SN-LiTFSI mixtures, both in liquid state, were combined and systematically studied in weight ratios of 5:0, 4:1, 3:2, 1:1, 2:3, and 1:4. For clarity, these mixtures were denoted as M_xS_y, where x and y represent their relative weight ratios. Polymerization of M_xS_y was achieved by adding AIBN initiator (1%) and PEGDA crosslinker (0.5%) at fixed molar ratios over MMA in the sample, followed by heating at 70°C for 12 hours. The liquid-state mixtures were homogenized using magnetic agitators. The polymerized sample was denoted as PM_xS_y, with PM_2S_3 specifically referred to as PMS for battery assembly unless otherwise noted. Additionally, a small amount of FEC (5 wt% of SN/MMA) was added to the electrolyte for LMBs assembly.

1.3 Battery assembly

Battery assembly was conducted in a glove box by injecting the homogenized liquid electrolyte into the coin and pouch batteries. The cathode was prepared by dissolving the PVDF binder in NMP and mixing it with the active materials (LFP, NCM811, and $LiCoO_2$) and Super P in a fixed weight ratio of 1:8:1. The mixture was homogenized into a slurry by intense stirring, cast onto Al foil, and dried under vacuum at 110°C for 24 hours before battery assembly. The loading densities of the active materials were 2-

2.5 mg cm^{-2}. The prepared cathodes were assembled with liquid-state electrolyte and Li anode to create full batteries. Polymer diaphragms were used to maintain the stability of the liquid-state electrolyte and improve reproducibility. Porous cellulose membranes are employed in the blocked battery cells due to excellent wettability with the electrolytes, while Celgard 2500 membranes are utilized in lithium symmetric, half, and full cells. The liquid electrolytes in the sealed batteries were polymerized using the same protocol mentioned above, resulting in the assembly of various battery types (blocked battery cell, Li symmetric battery, half- and full- batteries) for subsequent tests.

1.4 Electrochemical measurements

Electrochemical impedance spectroscopy (EIS), lithium transfer number (t_{Li^+}), cyclic voltammetry (CV), electrochemical floating, and linear sweeping voltammetry (LSV) measurements were performed using an electrochemical workstation (CHI660, CH Instruments, Inc., Shanghai). EIS measurements were conducted in a frequency range from 0.1 Hz to 1 MHz with an AC voltage amplitude of 10 mV. The t_{Li^+} was measured with a 10 mV DC polarization using the Bruce-Vincent method. CV measurements were performed at a scanning rate of 0.1 mV s^{-1}. Electrochemical floating experiments were conducted in a potential range from 4 V to 4.7 V vs. Li^+/Li for 300 minutes, recording the corresponding currents. LSV measurements were performed in the voltage range from 2 to 6 V vs. Li^+/Li at a scanning rate of 1 mV s^{-1}. Galvanostatic charge-discharge (GCD) measurements, Li stripping/plating measurements, and cycling tests of the batteries were conducted using a battery analyzer (LAND-

CT3002A, Wuhan LAND Electronics Co., Ltd., China). GCD measurements were carried out in the voltage range of 2.5-4.3 V at constant C-rates ranging from 0.1 to 5 C at 30°C. Note: 1 C was defined as 170, 200, and 200 mA g^{-1} for LFP, NCM811, and LCO cathode batteries, respectively. Li stripping/plating measurements were performed with a fixed area capacity (e.g., 0.1 mAh cm^{-2}) and varying current density from 0.1 to 1 mA cm^{-2}. Cycling tests of the coin batteries were conducted in the voltage range of 2.5-4.3 V at constant C-rates (0.5 or 1 C) at 30°C. Note: Battery batteries were not activated before the cycling test.

1.5 Structure Characterization

Surface morphology was characterized by scanning electron microscopy (SEM, Carl Zeiss AG, Germany). Samples from cycled batteries were protected with Argon during transfer to avoid damage. X-ray diffraction measurements were performed using a Rigaku Miniflex 600 diffractometer (Cu-Kα source) with a scanning range of 2θ from 10° to 70° and a scanning rate of 5° min^{-1}. Raman data was collected using a Zolix Finder 930 setup (laser wavelength 532 nm). XPS measurements were performed using a ThermoFisher ESCALAB Xi+ instrument with an Al Kα source and MAGCIS module (Ar as the ion source). Samples were prepared and transferred to the XPS setup without air exposure, using a glove box combined with the XPS setup. XPS data fitting was conducted using Avantage software. Small-angle X-ray scattering (SAXS) experiments were conducted with a Xenocs XEUSS 3.0 set up, equipped with a Cu K_α source (wavelength, λ =1.54 Å) and a Eiger 1 M detector (Dectris, Switzerland). The SN-LiTFSI liquid sample was sealed in a thin wall (a wall thickness of 0.01 mm and an

outside diameter of 1.5 mm) and quartz capillaries (Hilgenberg GmbH, Malsfeld, Germany) with epoxy resin, and all samples were measured in the vacuum chamber attached to the X-ray set up. The exposure time was 900 s, and all the measurements were performed at the room temperature. High-angle annular dark field scanning transmission electron microscopy (HAADF-STEM) and energy-dispersive X-ray spectroscopy (EDS) data were recorded using a JEM-1400Plus STEM/EDS microscope working at 200 kV. For the sample preparation, liquid M_2S_3 sample was first carefully drop-casted on the copper TEM grid, and then the sample was polymerized into PM_2S_3 by heated at 70℃ for 12 hours. All these operations were undertaken in argon-filled glove box. During the transfer to the TEM chamber, the sample was sealed in an argon atmosphere.

1.6 Mechanical Property Measurements

A nanoindenter (Step 700 Noise Control UNHT³, Anton Parr, Graz, Austria) was used to measure the elastic modulus and hardness of the elastomer electrolyte membrane. Hardness data were collected using a Shore Durometer Type C (LX-C, China) by preparing elastomer electrolyte membranes thicker than 6 mm. For the peeling test, elastomer electrolyte samples (5 mm wide and 20 mm long) were sandwiched between two Cu foils. Peel strength was measured using a micro material tester (HZ-1007C, Yiheng Instrument Co. Ltd., China). One Cu foil was peeled from the elastomer electrolyte membrane at a 180° angle at a constant displacement rate of 1 mm min^{-1}. The applied load was continuously measured, and force/displacement plots were generated. Each test was repeated at least three times.

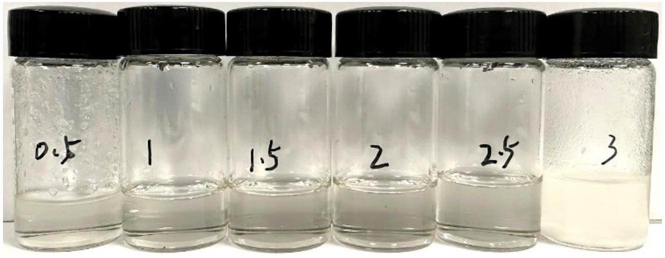

Fig. S1 Optical images of SN-LiTFSI with different molar ratios of SN and LiTFSI at room temperature.

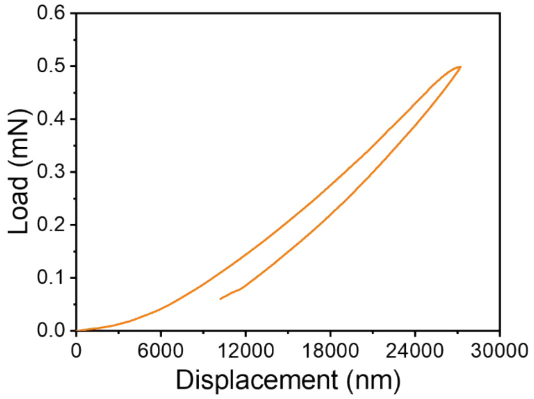

Fig. S2 Load-displacement curve of PM_2S_3 elastomeric electrolyte.

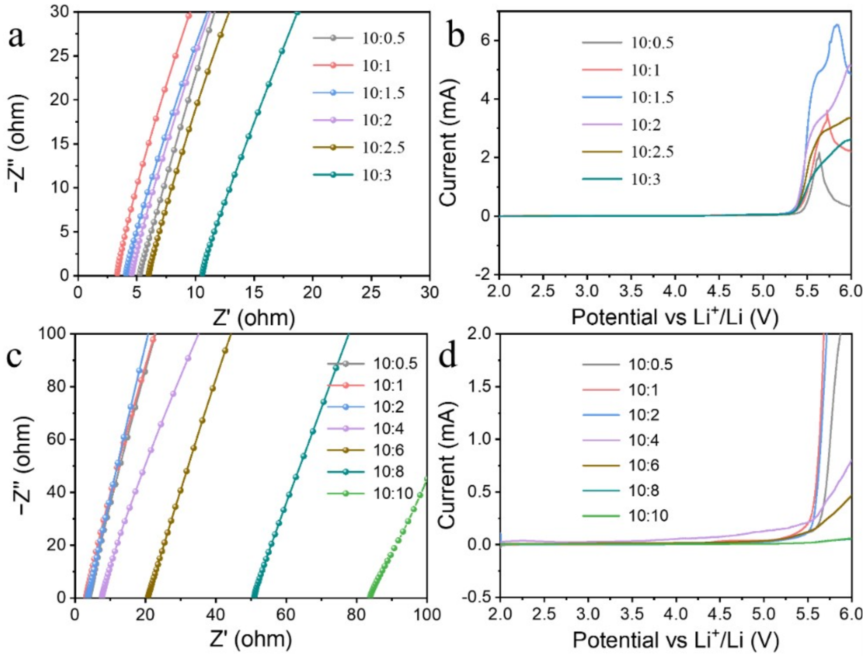

Fig. S3 (a) EIS and (b) Electrochemical window of solutions with varying molar ratios

of MMA and LiTFSI. (c) EIS and (d) Electrochemical window of solutions with varying molar ratios of SN and LiTFSI.

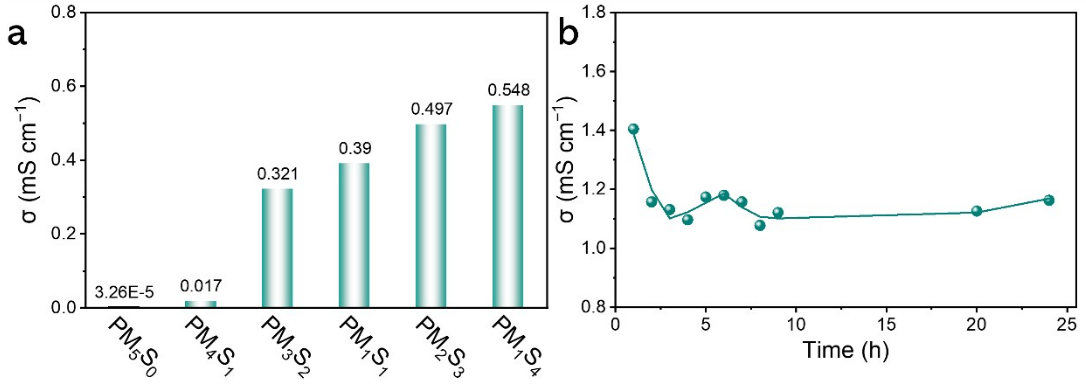

Fig. S4 (a) Ionic conductivity of PM_xS_y samples with different weight ratios of MMA-LiTFSI and SN-LiTFSI at 70°C for 12 hours. (b) Thermal evolution of ionic conductivity for PM_2S_3 with different heating times at 70°C.

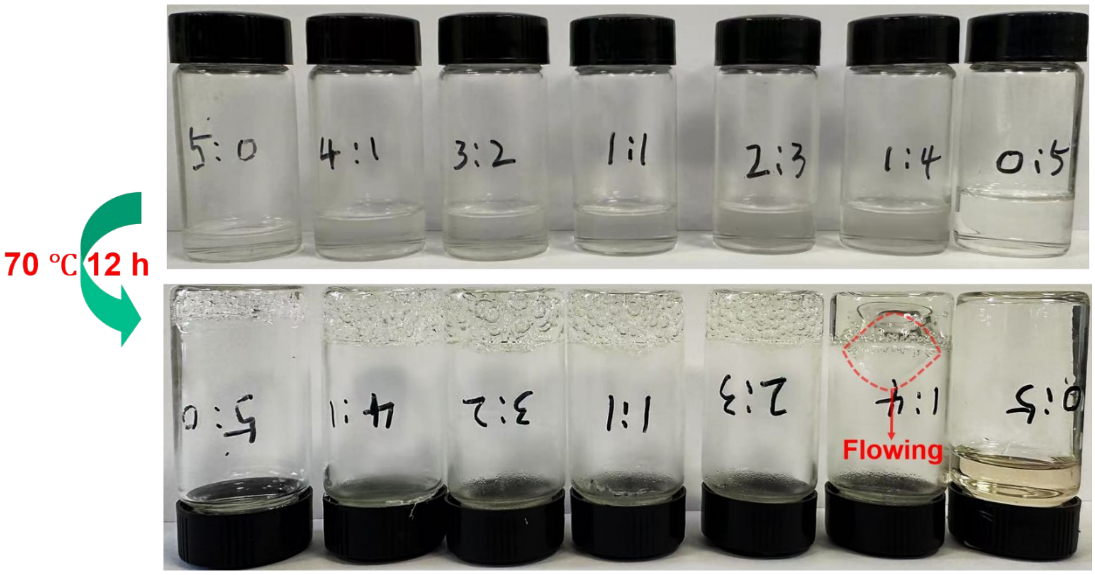

Fig. S5 Optical photographs of PM_xS_y samples with different weight ratios of MMA-LiTFSI and SN-LiTFSI at 70°C for 12 hours. The liquid-like feature of PM_1S_4 is marked with a dashed-line circle.

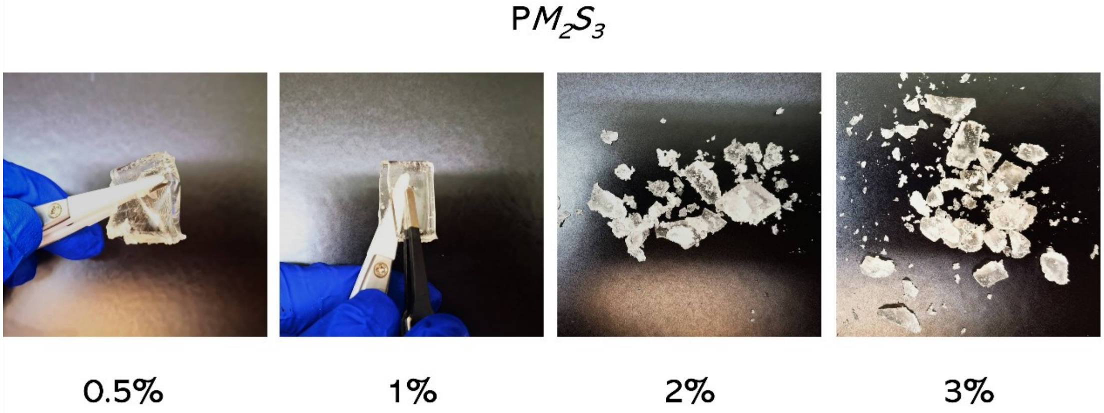

Fig. S6 Optical photographs of PM_2S_3 with different content of crosslinker (PEGDA).

A series of PM_2S_3 electrolytes was synthesized with varying PEGDA loadings (0.5%, 1%, 2%, and 3% molar ratios to MMA). A low PEGDA content (0.5%) resulted in a sparse PMMA network with low modulus, as evidenced by the easy bending of the electrolyte membrane (Fig. R6). Conversely, excessive PEGDA (>1%) increased structural rigidity, making the PM_2S_3 electrolytes brittle and prone to mechanical fracture under compressive stress (Fig. R6). To balance modulus and flexibility, a moderate PEGDA content (1%) was selected, enabling the electrolyte membrane to accommodate a high LiTFSI content for the 'polymer-in-salt' structure while maintaining good flexibility (as shown in Fig. 1 of the manuscript). Similar strategies have been employed in pioneering studies by Kim et al. for designing elastomeric electrolytes.[1]

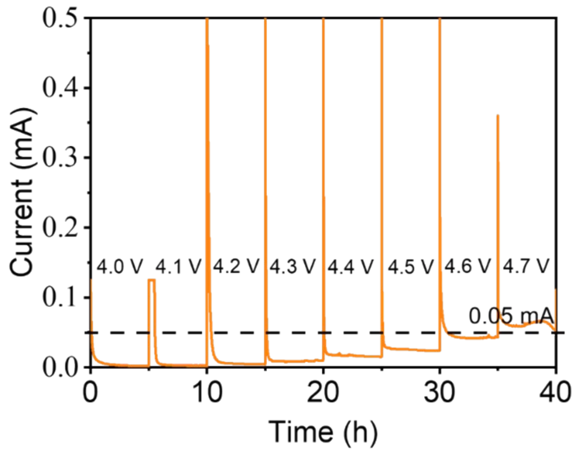

Fig. S7 The electrochemical floating experiment of NCM811|PM_2S_3|Li full batteries**.**

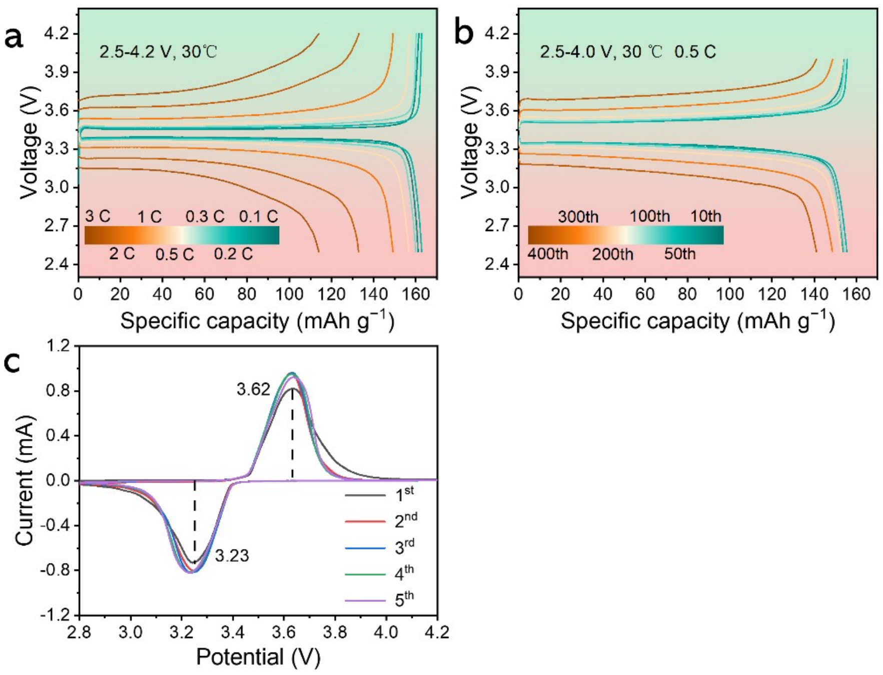

Fig. S8 (a) Charged/discharge voltage profiles of LFP|PM_2S_3|Li battery at different rate. (b) Charge/discharge voltage profiles of different cycles obtained from LFP|PM_2S_3|Li at 0.5C (30°C). (c) CV cures of LFP|PM_2S_3|Li.

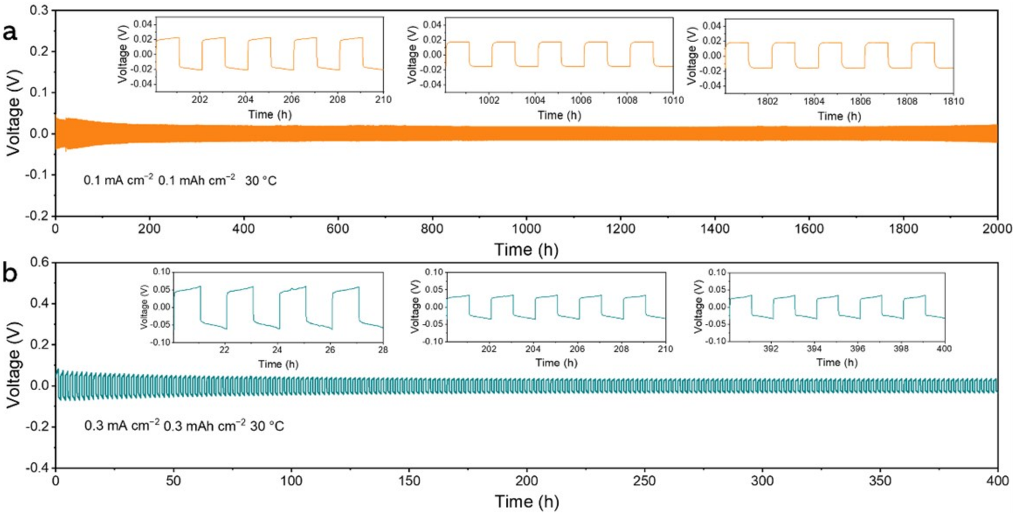

Fig. S9 Galvanostatic cycling curves of Li|PM_2S_3|Li symmetric battery under (a) 0.1 mA cm^{-2}, and (b) 0.3 mA cm^{-2} at 30°C. The typical amplified polarization voltage curves of the symmetric battery after different cycles are shown in the inset.

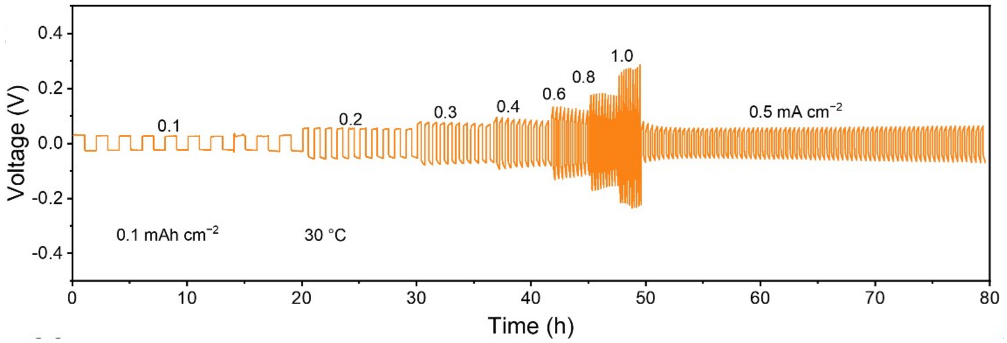

Fig. S10 Galvanostatic plating/stripping curves of the Li|PM_2S_3|Li symmetric battery under different current densities with fixed area capacity of 0.1mAh cm^{-2}.

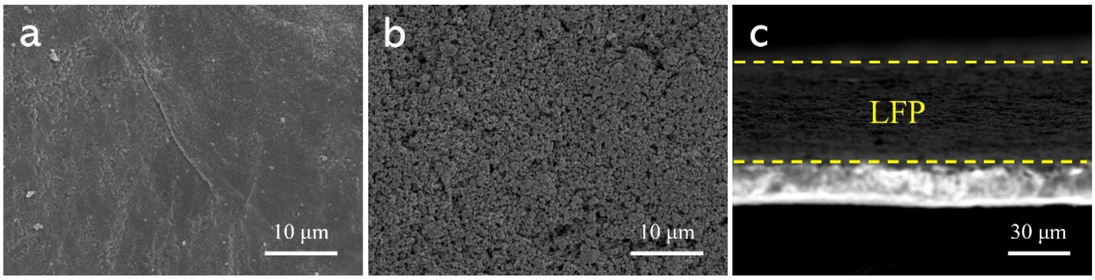

Fig. S11 (a) SEM surface images of Li metal of symmetric Li||Li batteries with PM_2S_3 after 2000 h cycling at the current density of 0.1 mA cm^{-2}. (b) surface and (c) cross-sectional SEM images of LFP of Li||LFP batteries with PM_2S_3 after 500 cycles at 1C.

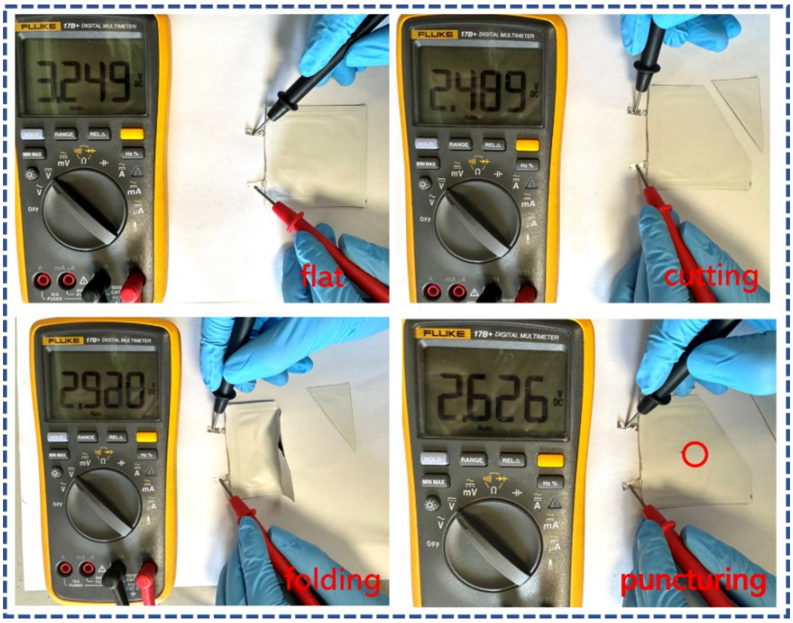

Fig. S12 The security tests by multimeter under extreme circumstances of NCM811|PMS|Li soft-pack battery

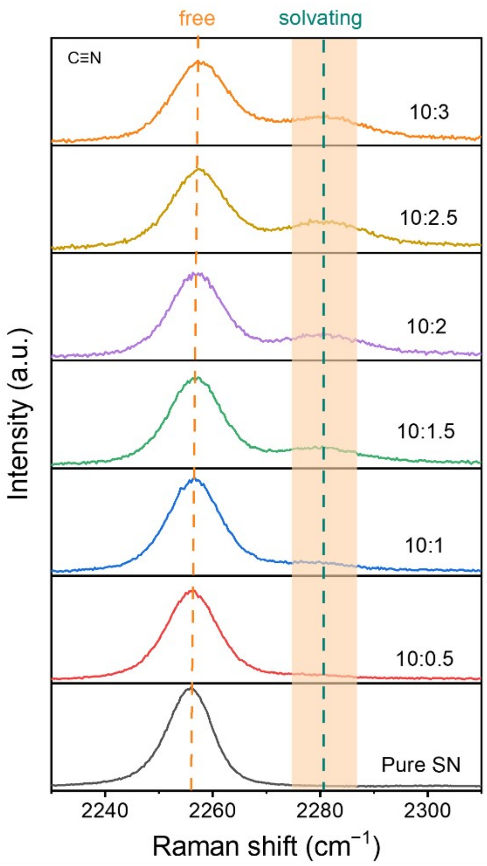

Fig. S13 Raman spectra of solutions with varying molar ratios of SN and LiTFSI.

To identify the solvated structure of the TFSI solution, Raman spectra of LiTFSI/SN solutions with varying lithium salt concentrations were obtained (Figure S13). The Raman spectrum of pure SN in Figure S13 shows a C≡N stretching band (v_2 mode) at 2258 cm^{-1}, originating from free SN molecules (i.e., not coordinated with Li^+).[1] As the molar ratio of SN to LiTFSI in the solution increases, another v_2 band appears at 2282 cm^{-1}, indicating the presence of Li^+ solvated SN molecules.[1]

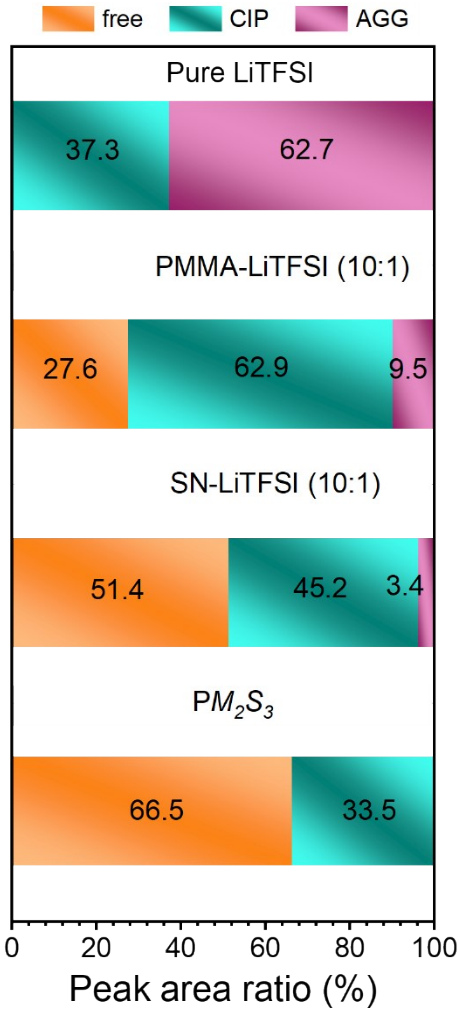

Fig. S14 Agglomerate structure of $TFSI^-$ in the PM_2S_3 and reference samples, determined by the relative area ratio of the fitted Fig. 5 (a) Raman peaks (i.e., free anions, CIPs and AGGs).

Reference

1. J. Han, M. J. Lee, J. H. Min, K. H. Kim, K. Lee, S. H. Kwon, J. Park, K. Ryu, H. Seong, H. Kang, E. Lee, S. W. Lee and B. J. Kim, *Adv. Funct. Mater.*, 2024, **34**, 2310801.
2. Y. Yamada, K. Furukawa, K. Sodeyama, K. Kikuchi, M. Yaegashi, Y. Tateyama and A. Yamada, *J. Am. Chem. Soc.*, 2014, **136**, 5039-5046.
